# Analysis and evaluation of peer group support for doctors in postgraduate training following workplace violence and aggression

**DOI:** 10.1192/bjb.2024.32

**Published:** 2025-04

**Authors:** Rowena Carter, Sharli Paphitis, Sian Oram, Isabel McMullen, Vivienne Curtis

**Affiliations:** 1 National Health Service Executive (formally Health Education England), London, UK; 2 South London and Maudsley NHS Foundation Trust, London, UK; 3 Institute of Psychiatry, Psychology and Neuroscience, King's College London, UK

**Keywords:** Violence, doctors, support, peers, workplace

## Abstract

**Aims and method:**

Workplace violence and aggression toward healthcare staff has a significant impact on the individual, causing self-blame, isolation and burnout. Timely and appropriate support can mitigate harm, but there is little research into how this should be delivered. We conducted multi-speciality peer groups for London doctors in postgraduate training (DPT), held over a 6-week period. Pre- and post-group burnout questionnaires and semi-structured interviews were used to evaluate peer support. Thematic analysis and descriptive statistical methods were used to describe the data.

**Results:**

We found four themes: (a) the experience and impact of workplace violence and aggression on DPT, (b) the experience of support following incidents of workplace violence and aggression, (c) the impact and experience of the peer groups and (d) future improvements to support. DPTs showed a reduction in burnout scores.

**Clinical implications:**

Peer groups are effective support for DPT following workplace violence and aggression. Embedding support within postgraduate training programmes would improve access and availability.

## Background

The effect of workplace violence and aggression on National Health Service (NHS) staff is significant, and causes a range of emotions such as anger, sadness, worthlessness, emptiness, fatigue and sleeping/eating disturbance.^1,2^ It can leave staff members questioning their competence and confidence,^3^ their organisation and their willingness to continue in the job.^3^ It can also result in mental health problems: in a Chinese cross-sectional study, 28% of healthcare staff working in public hospitals experienced post-traumatic stress disorder following incidents of workplace violence.^4^ Workplace violence and aggression is also associated with substantial cost to employers in terms of increased sickness, absence or legal action by the employee.^5,6^ Figures from the National Audit Office estimated that the direct cost of violence and aggression in the NHS is £69 million, with further costs likely as a result of staff retention, staff stress and burnout.^7^ In 2019, the ‘Understanding Career Choices in Psychiatry’ report^8^ identified violence and aggression from patients to staff as a key challenge to trainee retention.

Over the past two decades, clinical need and funding has driven violence and aggression prevention plans in healthcare. These have demonstrated variable success but, despite this funding and public health interest, there is nothing in the literature to suggest that violence and aggression perpetrated by patients toward staff can be eliminated from healthcare settings.^9–17^ It is therefore critical that staff support following workplace violence be prioritised. Although most studies looking at prevention recognise the need for trauma-informed staff support, the evidence behind how this should be provided is less certain.^18^

## Support following critical incidents

Single-session debriefing following traumatic incidents has been the subject of some controversy and, following a statement from the World Health Organization (WHO) advising against its use, is not currently offered. The WHO statement followed a 2002 Cochrane review^19^ suggesting that, at best, the intervention was neutral, and at worst, harmful. More recent literature has sought to address some of the criticism of single-session debriefing, stating that methodological differences and intended audience are important considerations when assessing the potential benefits/harms of this intervention.^20,21^

There are several models available to support healthcare staff following critical incidents (such as mass shootings, bombings, fire, drownings, terrorist acts, traumatic resuscitations and suicides), which have been evaluated across a range of front-line and emergency service staff.^18,22–25^ Many models offer some form of group event diffusion (also referred to as debriefing), which can happen immediately after the incident^24,26^ or within 72 h.^23^ All models suggest that follow-up is essential (i.e. not just a single session), with either group or individual sessions offered days to weeks after the event.^23,27^ Generally, the sessions are manualised, and commonly run through stages such as facts, thoughts, reactions, symptoms, education and normalisation.^28^ This type of guided event exploration and support is being used in many different forms, with evidence that they have a positive impact on staff in terms of improvements in staff well-being and psychological well-being, quality of life and resilience.^22,29,30^ However, there is much diversity in available practice, and there is little in the way of guidance to help employers understand the best model to support staff following traumatic incidents in the workplace. And, despite the high global incidence of workplace violence and aggression toward physicians,^31^ there remains a paucity of literature evaluating staff support specifically for doctors in postgraduate training (DPTs) following incidents of workplace violence and aggression.

## Peer support

Peer support programmes are defined by Cyr et al^32^ as a supportive relationship between individuals who have experienced adverse events, providing emotional and social support, encouragement and hope. Rather than being single sessions or single sessions with a follow-up, they are ongoing programmes to deal with and manage issues as they arise. In 2020, Anderson et al^33^ reviewed the use of peer support following critical incidents and found eight studies across emergency services, with results in favour of peer support, reporting improvement in sick leave, mental health and reduction in suicide rates.^33,34^ In their paper, Carleton et al^35^ found that peer group engagement reduced feelings of stigma toward self and others.

With the exception of peer support,^36,37^ other methods of staff support have not been specifically evaluated following incidents of workplace violence and aggression in a healthcare setting. Furthermore, we found no evaluations of any model for the specific support of DPTs following workplace violence and aggression. For this reason, we chose to offer and evaluate the use of peer support groups for DPTs following incidents of violence and aggression. We also wanted to understand the current experiences of DPTs following workplace violence and aggression, to help build support and interventions in the future.

## Method

### Study design

We designed a cross-sectional peer group intervention for all DPTs in the London Deanery, as defined by Health Education England (now part of NHS England). Groups ran from February 2021 to April 2021, and a mixed-method approach was used for analysis.

### Inclusion and exclusion criteria

Health Education England is an executive public body under the UK Department of Health and Social Care, which provides leadership and coordination for the education and training of the NHS workforce in England. DPTs are medical professionals who have completed their undergraduate medical school examinations and are currently working as doctors within a training programme overseen by Health Education England. Inclusion criteria for the study are listed below.

- Participant was a DPT within a London training scheme. All specialities were eligible (including surgical, medical, psychiatry, paediatrics, radiology, histology, anaesthetics and intensive care unit, general practice, emergency medicine, obstetrics and gynaecology, public health, ophthalmology and foundation training), as were all grades from foundation training (FY1 and FY2), Core training (ST1–ST3) and speciality training (ST4–ST8). DPTs could be working in either the community or hospital setting.
- Participant was a DPT currently undertaking time out of training programme (e.g. maternity leave, research, sick leave).
- Participant was able to attend the group sessions weekly for a period for 6 weeks (with the understanding that apologies could be made for urgent/unforeseen circumstances).
- A personal experience of workplace violence or aggression was not required.

Excluded from the study were any non-medical health professionals, anyone not considered to be DPT by the definition provided by Health Education England (e.g. consultant) or anyone who left training more than 3 years before the commencement of the group.

### Recruitment

Recruitment for participants was managed in two ways: (a) DPTs participating in a survey on experiences of workplace violence and aggression could provide an email address to be contacted with further information on the peer groups; and (b) Health Education England sent out an email informing DPTs of the peer group, with an email address to contact for further information.

All of those who expressed interest were contacted and provided with an information sheet. Participants who completed the intervention were offered an opportunity to take part in a semi-structured interview (see Supplementary Appendix 1 available at https://doi.org/10.1192/bjb.2024.32).

### Structure and running of the groups

Sessions ran for 60 min, using the following format.

- Introduction (5 min): An informal greeting and welcoming to/back to the group.
- Case presentation (15 min): Each week a different DPT opted to present a case that had impacted them involving violence and aggression, they were asked to omit identifying patient details, to speak from the ‘I’ in terms of the impact on them, and to give details of the event as well as any relevant surrounding details in the case for context. This is a similar model used in Balint group discussion, where the group listens to the case presented and are then allowed some time to ask questions and clarify facts.
- Discussion (25 min): DPTs then discussed the case and linked it to experiences of their own – this was a free discussion with an emphasis on the emotional impact on the trainee, and the facilitator provided guidance when the discussion became operational.
- Coping strategies (10 min): Each week a new coping strategy was explored and feedback was provided for the previous week's strategy, with regards to usefulness for the DPTs – the strategies explored were concept of coping strategies, mindfulness, journaling, self-monitoring, reframing and setting goals (for weeks 1–6, respectively).
- Mindfulness practice (5 min): Each week DPTs alternated between two guided mindfulness breathing tasks.

Two groups were run over Microsoft Teams version 4.12 for Windows (virtually) for a 6-week period. One session was held at 14.00–15.00 h on a week day, the second was held at 20.00–21.00 h on a weekday.

### Data collection

The Copenhagen Burnout Inventory^38^ was administered before commencement of the group and at 2 weeks following completion of the final session. The inventory asks 19 questions and examines burnout across three domains (personal, work related and client related); it uses a five-point scale from always to never. The inventory has a Cronbach's alpha of 0.85–0.87 (indicating high internal consistency) and has been translated into multiple languages, being used in burnout research across the world.^39^ It is used in the UK by the annual national trainee survey run by the General Medical Council.

Semi-structured interviews (Supplementary Appendix 1) were conducted 2 weeks after completion of the peer groups. Interviews followed a topic guide and were transcribed by R.C. Identifying details were removed from transcripts. The questions explored DPTs experiences of workplace violence and aggression, support in the organisation and both negative and positive experiences of the peer groups, including questions relating to accessibility. Questions for suggested future improvements were also asked.

### Data analysis

**Fig. 1:**
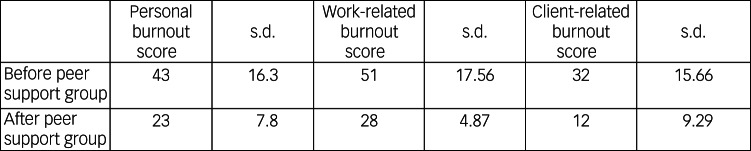
Average group burnout scores, as per the Copenhagen Burnout Inventory, before the peer support groups and then between 2 and 3 weeks after peer support groups had finished.

Descriptive statistics have been used to describe the difference in burnout scores before and after the peer groups (Fig. 1). On the Copenhagen Burnout Inventory, a score of 0–25 represents ‘no burnout’, 26–50 represents ‘mild burnout’, 51–75 represents ‘moderate’ burnout and 76–100 represents ‘severe’ burnout.

Thematic analysis of semi-structured interviews has been conducted following the Braun and Clarke^40,41^ model for thematic analysis. Data was coded by allocating excerpts of text to thematic codes representing meanings in the data. Related codes were combined to create themes, which were reviewed and organised until a master list of thematic codes was created. Codes and data were checked and confirmed by a second researcher.

### Ethical approval

The study received full ethical approval by the Research Ethics Office at King's College London (reference number HR/DP-21/22-26273f) before commencement of the study. All participants read information sheets regarding the study and were given the opportunity to speak with the group facilitator before providing informed consent to join the group. Participants could request withdrawal of their data up until 1 month after the interviews.

## Results

A total of 11 DPTs were recruited for the peer groups (one group had four DPTs and the other had seven DPTs). One DPT dropped out of the first group after two sessions as they were unable to continue because of the timing of the group. A second DPT dropped out of the second group after three sessions, because of planned maternity leave. A total of nine DPTs completed the 6-week course, with several missing at least one session because of work commitments. Nine participants completed the pre-group burnout questionnaire, and six completed the post-group burnout questionnaire.

All participants identified as female. Their training grades ranged from ST1 to ST6, and the specialities represented were psychiatry, general practice, orthopaedics, maxillofacial surgery, accident and emergency, medical core training, infectious disease and Acute Care Common Stem (ACCS) core training. Other than the general practitioner, all of the DPTs were working in hospital settings. Four of the DPTs had experienced previous or current psychological support and therapy in relation to the incident of workplace violence and aggression. Although experience of workplace violence and aggression was not necessary to attend the peer group, all DPTs had some personal experience of workplace violence and aggression perpetrated by patients. Six of the seven DPTs also experienced a lack of support or bullying from staff members following the incident.

### Descriptive statistical analysis

The average burnout scores decreased across all domains after the peer groups. Personal burnout changed from mild burnout before the peer groups started, to no burnout after the peer groups finished. Work-related burnout changed from moderate burnout before the peer groups started, to mild burnout after the peer groups finished. Client-related burnout changed from mild burnout before the peer groups started, to no burnout after the peer groups finished.

### Thematic analysis

Seven DPTs took part in a semi-structured interview. Thematic analysis revealed four organising themes: (a) the experience and impact of workplace violence and aggression on DPTs, (b) experiences of support following incidents of workplace violence and aggression, (c) impact and experiences of the peer groups and (d) future improvements to support.

#### Theme 1: the experience of workplace violence and aggression and its impact on DPTs

All DPTs described having personal experience of violence and aggression in the workplace; experiences ranged from physical assault to multiple episodes of verbal or passive aggressions. DPTs also spoke about the witnessing incidents of violence and aggression in the workplace (Supplementary Data 1), and concerns that they were put at unnecessary risk of violence and aggression in their role (Supplementary Data 2).

DPTs spoke about the impact workplace violence and aggression had on their personal and professional lives. All DPTs felt that this impact was negative and that they had been limited as a result. Personal impacts included depression, anxiety and flashbacks. Professional impacts including leaving the speciality, moving deaneries, taking time off, doubting professional competency and burnout (Supplementary Data 3).

‘I now don't like working on the wards at all, I feel very ‘aware’ of the patients, if that makes sense, like when they move around me or near me. I am very carefully what I say to them, the other thing is that I now don't trust that other staff will ‘have my back’ (Participant 1).

Although patient-on-staff violence and aggression was the focus of this project, many DPTs commented on the impact of staff-on-staff (Supplementary Data 4) bullying and harassment as something that was both common and had a significant impact on their well-being. Experiences ranged from judgement, dismissiveness and bullying (being placed on a difficult rota, referred to Health Education England and prevented from progressing in training). This was often triggered by a patient-on-staff incident of violence and aggression, but could also occur *de novo*.

‘I would say colleague on colleague is worse, it is more frequent and more damaging. I see that a lot, people speaking behind others backs, saying x/y/z doesn't work hard, being critical if people have to leave for childcare or are sick. I find it very draining’ (Participant 6).

#### Theme 2: DPTs’ experiences of support following incidents of workplace violence and aggression

DPTs spoke about the importance of immediate staff response as well as subsequent support in the weeks following the incident, and the resulting impact this had on their well-being. Most DPTs commented on the lack of support in the immediate and medium term following the incident of violence and aggression. Several sought their own therapy and support (Supplementary Data 5). In some cases, DPTs experienced staff-on-staff bullying and victimisation (Supplementary Data 6), with DPTs having their jobs and positions threatened after reporting violence and aggression to a senior staff member or colleague. All DPTs said that they approached their supervisor informally; only one used Datix (a national formal incident reporting tool). DPTs felt that their supervisors did not know how to support them.

‘The worst thing was the reaction to me afterwards where I was made to feel a burden on the team. I was then labelled as a problem trainee and from that point onwards I was bullied by my seniors and the rota coordinator who made my life very difficult’ (Participant 3).

‘This incident led to a series of bulling events and lack of support from well-being lead and other staff with seniors accusing me of not pulling my weight, faking illness, being a poor communicator. After a few weeks they threatened my job and career going forwards’ (Participant 5).

DPTs identified several barriers to currently accessing support, and many of these were centred around the knowledge and reaction of their senior when the incident of violence and aggression was reported (Supplementary Data 7).

‘It needs a big change, like more staff, to make things really better. I can't see that the well-being group stuff makes much difference because no one really has time to go, I also have heard people call it “fluffy” or similar, implying weakness or that it's not really valued, it makes me feel funny about going to things like well-being because it's not really seen as important. So I guess that needs a cultural change’ (Participant 2).

#### Theme 3: the impact and experience of the peer groups on those in attendance

The DPTs were positive about the access to the peer groups, reporting that the virtual format and session length had worked well for them (Supplementary Data 8). All DPTs agreed that they understood what the content and layout of the session would be, how to access further support if needed and when/how the sessions would end.

The DPTs identified several advantages of attending the peer groups. Many of them commented that they liked that the groups comprised peers (those who could understand the job) who were not direct peers, as it provided an understanding but maintained a degree of anonymity. DPTs said that through the groups they felt validated, that their experience was normalised, that it was a reflective and safe space to share experiences and that they were glad for the experience (Supplementary Data 9).

‘Having meaningful conversation with people who know about medicine but not in the same work place as you was really helpful and felt unique. Hearing the experiences of others and people offering their own perspective and support felt really helpful and supportive. I felt positive and better after I presented’ (Participant 7).

Five of the seven DPTs commented that they liked the mindfulness space at the end of the group. Many also commented that they enjoyed learning a new skill: the journaling/diary keeping exercise was mentioned specifically by three DPTs as something they intended to continue, as was the mindfulness.

‘I liked the idea of journaling and also mindfulness, I have been doing mindfulness’ (Participant 2).

DPTs also identified some difficulties with attending peer groups, some expressed that a group environment might not suit all DPTs and commented on the importance of the right facilitator (Supplementary Data 10). DPTs were also less sure about the timings of the groups, with many commenting that it was hard to fit in to their normal working week (despite one of the sessions being conducted out of hours to help facilitate this).

‘I am not sure the groups would be for everyone, it's hard to know and I think we were all confident but I can imagine if there is someone in the group who doesn't want to be there or who is really dominant it might make the group feel quite toxic’ (Participant 2).

All DPTs said that they would like to continue the peer group sessions, and most commented that they felt these should be integrated into the working week to facilitate attendance. Many also felt that peer groups should be something that was offered proactively rather than something you might opt to attend when an incident at work happens.

‘It would maybe have been better if there was a time found for this within work hours, but then it would have to be something everyone attends otherwise you would feel singled out as someone struggling so maybe it wouldn't work’ (Participant 6).

#### Theme 4: future improvements to support

DPTs highlighted issues and problems with the systems that needed improvement, they suggested increased security staff, more security equipment, increased staffing in general as well as resource to decrease the likelihood of incidents occurring in the first place (Supplementary Data 11). They also commented on the need for a better system approach for identifying DPTs who need support.

Almost all DPTs highlighted the importance of colleague and senior staff responses following their experience of violence and aggression, and all felt that improvements needed to be made in the way that senior staff and colleagues supported DPTs following incidents of violence and aggression (Supplementary Data 12).

‘I think seniors knowing/understanding how to react and where to direct trainees after these things happen would be helpful. I think having a space to discuss this with colleagues is helpful too but I would prefer if it wasn't with direct colleague’ (Participant 2).

DPTs felt that access to support was challenging and that there was not enough emphasis placed on well-being provision, psychological support or other support structures available. They suggested that this was partly educational/induction need, partly a problem with the system identifying those who need support and partly a problem with senior staff who did not understand where to signpost DPTs for additional help.

‘I think meeting with your line manager would be helpful to receive some support and validation and to identify if any further signposting were needing the after care is somewhere that a lot of improvement needs to happen. I think we could probably provide support in house, the governance structure within the hospital, our Datix reviewed on a weekly basis and the line manager can pick up on these easily and could offer an interview and support’ (Participant 4).

DPTs overall felt that the peer groups were beneficial to them, and most felt that they should be embedded within the programmes and timetables so as to facilitate attendance.

‘I think the answer needs to come externally, I think there needs to be a safe place for trainees and it has been really helpful to understand the peer group model because I think it works well for this. The anonymity of being with trainees from different specialties was helpful and also talking with trainees rather than someone in an “official” capacity made it more comfortable to share. I think there needs to be dedicated time for sessions like this, which occurs within your normal work day’ (Participant 3).

## Discussion

The need for post-incident staff support following work-place violence and aggression has been highlighted by policy makers;^38^ however, the barriers to access, lack of support from organisations and underreporting of incidents make it hard to identify staff who need support. Evidence suggests that well-timed and appropriate support following critical events (which include, but are not exclusively, violence and aggression) can help staff retention^42^ and emotional well-being,^9^ and decrease the impact of the event.^43^ Peer support (in different forms) has favourable evidence post-critical events, with studies reporting improvement in the time taken by staff on sick leave, improvement in mental health (fewer symptoms of post-traumatic stress disorder), lower suicide rates and decreased feelings of stigma in participants.^33–35^

The evidence in this study suggests that virtual peer groups may be a helpful intervention for DPTs from all specialties who opt to take part following workplace episodes of violence and aggression.

DPTs who attended virtual peer support groups reported that the groups provided a means of validating their experiences, and a supportive and non-judgemental environment. All DPTs who attended said that they would return for more sessions if these were made available. Holding the sessions online improved the accessibility of the groups and provided DPTs with ‘anonymity with understanding of the profession’. The use of virtual peer groups has been found to be helpful, supportive and accessible in previous studies.^44,45^ The importance of good facilitation was highlighted, as was the small group sizes.

Our thematic analysis highlighted the importance of the initial colleague and senior staff response following the incident of violence and aggression, and it appears that the impact of the event can be mitigated if there is a supportive response; however, when the response is either unengaged, unsupportive or sets into motion events that, lead to bullying then the impact of the initial event escalates and can have dire consequences for the DPT (such as leaving the deanery or the speciality, as well as ongoing post-traumatic stress symptoms and anxiety). This has also been found in the literature.^36,46^ DPTs are especially vulnerable to these effects because they require their supervisor to allow them to progress within their training programme.

This analysis has highlighted that supervisors are the default reporting mechanism for DPTs following workplace violence and aggression, and their response is critical. However, some are far from validating, do not know where to signpost DPTs for external support and, in the worst cases, the DPT is bullied by their supervisor (or supervising organisations) after reporting the incident. In addition, DPTs feel disempowered to attend well-being and psychologically supportive groups or interventions for fear of being seen as ‘weak’ or ‘not resilient’. This creates the perfect storm, whereby DPTs feel isolated, victimised and unsupported. The experiences of the DPTs who took part in the peer group highlights a toxic and unhealthy organisational culture, as described by the systemic theory, that promotes competition and high stress, and results in lack of support and communication. The power dynamics and workplace belief about what makes a ‘good employee’ warrant further exploration. There also appears to be a lack of physical and emotional safety within these situations, highlighting the importance of the attachment theory in the workplace and the need to feel listened to, accepted and validated. Examining the values, norms and expectation within the NHS might help identify some of the factors contributing to aggression, as well as exploring the power dynamics, role ambiguity and the impact of workload on cultural expectation. The need for training and educating senior staff and those in support roles appears urgent.

DPTs expressed that they would like some form of support/peer group or safe space to discuss these issues embedded into the programme. They recognised that currently, many well-being facilities and sessions are not well attended, and that there is stigma attached to attending these that this might lead to further bullying or victimisation. Currently, Health Education England offers a support service, the professional support unit, which can provide individual psychological therapy as well as peer groups and career guidance. However, this involves the DPT proactively seeking this support, and they may not feel empowered to do this, especially if their supervisors are not aware of the service. Should more support be provided at the Trust level? Again, this feels less than ideal, as DPTs often move through Trusts as part of their training schemes and risk being lost to follow-up as a result. In this study, some DPTs felt that this function of support and reporting of incidents should be held outside of Health Education England and the Trust, for fear of reprisal upon reporting incidents.

DPTs wanted to see a change in the current systems and provisions, and wanted better protection from incidents happening in the first place, embedded support structures, better education for staff on the support available, and recognition and education about the potential impact on DPTs. It should be also considered that no two DPTs are the same, and no two have the same experience; the response to violence and aggression needs a nuanced approach.

### Study limitations

The lack of control group in this study makes it hard to draw inferences from the reduction in burnout of the DPTs over time: burnout is dependent on multiple factors, and the time between data collection points may have been sufficient enough to see a reduction. There is also some uncertainty as to whether burnout is truly being measured here, given the cross over with anxiety, depression and job dissatisfaction.

The sample size was small, all female and a self-selected cohort, and, although it was not a requirement for the DPT to have experienced workplace violence and aggression to attend, all those who opted to take part had experienced violence or aggression incidents in the workplace. The outcome of this study cannot be generalised to all DPTs, particularly not to those who do not have experience of workplace violence and aggression.

Finally, there is the potential for response bias, as semi-structured interviews were conducted by the same researcher who facilitated the groups.

In conclusion, there is a recognised need to support healthcare staff who experience workplace violence and aggression. Barriers include underreporting, workplace culture, lack of knowledge among supervisors and poor organisational resourcing. Embedding support within doctoral training programmes could help to mitigate these barriers. Peer support groups may be a helpful intervention for this group.

## About the authors

**Rowena Carter** is a Clinical Fellow at the National Health Service Executive, London, UK and a ST7 Psychiatry Trainee at South London and Maudsley NHS Foundation Trust, London, UK. **Sharli Paphitis** is a researcher in the Section for Women's Mental Health at the Institute of Psychiatry, Psychology and Neuroscience, King's College London, UK. **Sian Oram** is Head of the Section of Women's Mental Health at the Institute of Psychiatry, Psychology and Neuroscience, King's College London, UK. **Isabel McMullen** is a consultant psychiatrist at South London and Maudsley NHS Foundation Trust, London, UK and Lead for Wellbeing at the National Health Service Executive, London, UK. **Vivienne Curtis** is a consultant psychiatrist at South London and Maudsley NHS Foundation Trust, London, UK, a visiting senior lecturer at the Institute of Psychiatry, Psychology and Neuroscience, King's College London, UK and the Associate Academic Dean and Head of the School of Psychiatry, National Health Service Executive, London, UK.

## Supporting information

Carter et al. supplementary material 1 Carter et al. supplementary material

Carter et al. supplementary material 2 Carter et al. supplementary material

## Data Availability

The data that support the findings of this study are available on request from the corresponding author, R.C. The data are not publicly available due to their containing information that could compromise the privacy of the research participants.
