# Supplementary material for: Analysis and evaluation of peer group support for doctors in postgraduate training following workplace violence and aggression
Source: BJPsych Bull. 2024 May 16;49(2):78–85. doi: 10.1192/bjb.2024.32 (PMC12014390; doi:10.1192/bjb.2024.32)
Supplement: Carter et al. supplementary material 1 — Carter et al. supplementary material [file S2056469424000329sup001.docx]

**Appendix 1**

**Semi-structured interview**

**Background**

***Q1. What level of training are you currently in and what speciality?***

***Q2. Are you working in a hospital or community setting currently?***

***Q3. How do you currently feel about your current job?***

**Experience of Violence and Aggression at work**

***Q4. Have you had personal experience of violence or aggression at work?***

***Q5. Have you witnessed violence or aggression at work?***

**Engagement Barriers and Support**

***Q6. Did you have any difficulty engaging in the Peer Support Group?***

**Support**

***Q7. What other support have you been offered with regards to your experience of Violence and Aggression in the work place***

***Q8. Have you had any time off work or negative impact as a result of your experience of Violence or Aggression at work?***

***Q9. What do you feel would have been helpful following the experience of Violence or Aggression at work?***

**Positive qualities of peer group/Impact**

***Q10. What does positive impact or qualities mean to you?***

***Q11. Using your definition do you feel that peer groups have positive qualities or impacts***

***Q12. Did you find the group approachable?***

**Programme content/ending/set-up**

***Q13. Did you understand the content and purpose of the sessions?***

***Q14. Did you understand the set up of the group?***

***Q15. Was it clear about the ending of the groups? Was it clear where further support and help could be accessed?***

**What was helpful**

***Q16. What was helpful about the peer support group***

**What was unhelpful**

***Q17. What was unhelpful about the peer support group***

**Ways to improve the Programme**

***Q18. In what ways could YOU help yourself better cope emotionally that you have not yet identified?***

***External support***

***Q19. What support mechanisms does your employer have in place to assist you in being aware of, and coping with, the potential dangers of as a result of the work you do?***

***Q20. If you could suggest 3 changes that could be implemented at your workplace to improve the emotional safety, coping and resilience of violence and abuse, what would they be?***

**Current programme**

***Q21. How would you improve the current programme?***

**Any Further Input**

***Q.22 Is there anything you think we have not covered that you think we should have covered, or that you think is important for us to discuss?***
