# Supplementary material for: Analysis and evaluation of peer group support for doctors in postgraduate training following workplace violence and aggression
Source: BJPsych Bull. 2024 May 16;49(2):78–85. doi: 10.1192/bjb.2024.32 (PMC12014390; doi:10.1192/bjb.2024.32)
Supplement: Carter et al. supplementary material 2 — Carter et al. supplementary material [file S2056469424000329sup002.docx]

**Supplementary data**

**Supplementary data 1) - Witnessing incidents of violence and aggression in the workplace**

‘On short stay ward, we had someone who wanted some medication that we couldn’t prescribe, they started shouting at me on the ward and then when I turned to leave he hit me’ (Participant 1)

‘She turned to me and strangled me, I couldn’t scream or make a sound, I thought she was going to kill me’ (participant 2)

‘From patients they mostly complain about long waiting times, about the service, about not getting the right advice or having the advice change and not been given a reason, they can get quite nasty at times’ (participant 6)

‘I have seen people shouting and being abusive, I would say that is every shift’ (participant 4)

**Supplementary data 2) - Unnecessary risk of violence and aggression in role**

‘The patient had a security guard with her. I went into her bed space to speak to her, at this point the security guard left with her nurse and I was alone with her (participant 2)

‘The nursing staff saw and didn’t do anything to help’ (participant 1)

**Supplementary data 3) - Professional impacts of workplace violence and aggression**

‘I dreaded my shifts and it made coming into work really stressful’ (participant 2)

‘I changed jobs, I mean specialities’ (participant 3)

‘I went back before I was ready and had bad nightmares and struggled with hyper-viligence and anxiety’ (participant 5)

**Supplementary data 4) - Impact of staff-on-staff violence and aggression**

‘I think this question should really also cover aggression between staff members, I have seen a lot of that too, lots of shouting, criticising and backstabbing, to be honest. It make’s you wonder what they say behind your back’ (participant 1)

‘Another trainee (in the same job) I witnessed being bullied by the staff, she had a disability which meant that she was unable to perform tasks at the same speed as others, they said she was lazy, the nursing staff made comments about her’ (participant 5)

**Supplementary data 5) - Therapy and support**

‘The datix set lots of support processes in motion, I was also offered psychology which I took and did 2 sessions with the psychologist. I felt the attention was overall positive’ (participant 2)

‘I have just finished a period of CBT prior to starting the peer groups through occupational health’ (participant 4)

I wasn’t offered anything, I went for counselling personally which I found myself (participant 3)

**Supplementary data 6) - Experienced staff on staff bullying and victimisation after reporting workplace violence and aggression**

‘I was crying and the charge nurse just said ‘oh what’s wrong’ just so cold, she then told me it was ‘part of the job’ and I should be more resilient to this kind of thing. I thought ‘really, being hit on the head is part of the job? Then I don’t want this job’. Then she accused me of winding him up or upsetting him, months later when I told my clinical supervisor about it he didn’t give any sympathy, he told me off for not doing a datix. I just could not have felt less supported if I am honest with you (participant 1)

‘None of the staff supported me or helped, I felt very alone after this and I cried. No one said I should press charges and there was an expectation that I continue to treat this man. I told my consultant and my educational supervisor; I was made to feel that this isn’t a big problem and I should move on’ I felt no one stepped in to help me when it happened and I was really let down by colleagues (nursing and medical)’ (participant 6)

**Supplementary data 7) - Barriers to accessing support**

‘I just wish people were more understanding, its important who you go to for help, you cant trust everyone to react in the same way or do the right thing by you’ (participant 3)

‘Supervisors being more understanding or helpful. I don’t really know, it feels like the whole culture is the problem so how do you fix that?’ (participant 1)

‘I was not offered any additional support, I was told I could go home that day and have the following day off but this wasn’t really adequate. I felt that if I went home then it would be more stressful because the work load was so high, I was also worried (as a locum) I would not get paid if I went home or didn’t come in’ (participant 7)

‘The main barriers have been the seniors, when I reported what happened and had time off because of it the seniors began bullying me, they bullied me to return to work and then when I came back they were unkind, they called me work shy, they changed my rota to impossible hours and gave me more work than the other trainees’ (participant 5)

**Supplementary data 8) - The access to the peer groups**

‘No problems, I liked doing it online, I liked doing it after work as I felt it gave me a different space to do this I, I wouldn’t want to do the group and then go back to work, that would be hard’ (participant 2)

‘No issues, was easy to engage with, I found the time worked for me. I liked doing virtual, I am not sure how you would do this if it wasn’t virtual’ (participant 4)

‘As I get back to clinical work it would be very hard for me to take time to come to a peer group in the week’ (participant 5)

‘I was very grateful you offered an out of hours session or I would not have been able to come, I find that anything we try to do for ourselves (including mandatory training) is often expected to take place outside of clinical time’ (participant 6)

**Supplementary data 9) - Advantages of attending the peer groups**

‘Peoples reactions to your experience and the response of others going through it with you in a safe space, processing things. I felt I was able to be vulnerable in this group and cry, I couldn’t do that if they were my direct peers’ (participant 2)

‘I think it was really nice to share amongst a small number of people who understand your position. I felt the small number meant we made friendships rather than just colleagues, you could relate to their problems which were similar’ (participant 3)

‘I felt validated by others. It was also good to hear that I wasn’t alone in these experiences, that others had the same. For a while I felt like it was just because of me, because I hadn’t responded properly, if I was better with patients it would have turned out differently. I didn’t feel I could discuss these feelings with colleagues for fear I would seem weak or unable to cope’ (participant 6)

**Supplementary data 10) - Difficulties with attending peer groups**

‘I guess I wondered what would happen when we had all told our stories, like where would it go from there. Maybe people had more than one thing happen I suppose, so perhaps that was the plan but it was limited to 6 sessions anyway. I think I felt a bit sad when it ended’ (participant 1)

‘I found it really worrying to hear the experience of those who had been bullied when they went to get help, it made me worried about the NHS and the culture of seniors and other colleagues towards others who are having difficulty and it highlighted to me the idea of having problems was a bad thing or a burden to staff rather than something that should receive support. In that way I guess at times it made me feel hopeless’ (participant 2)

**Supplementary data 11) - Systems that needed improvement**

more security or people who can step in and help’ (participant 1)

‘I think that if there was more structured conflict resolution that would have been helpful. A mediated discussion and something more formal, following this having someone check whether I need emotionally support or not. That would have been helpful. Follow-up is really important and no one did this’ (participant 3)

‘a lot of de-escalation and breakaway training, that would be great’ (participant 4)

‘Some acknowledgement of the event, support in pressing charges, people actually agreeing that zero tolerance is zero tolerance, this patient could be told to go elsewhere for treatment’ (participant 6)

‘I would have wanted something more structured or formal, a sit down with a supervisors, a pathway laid out about what could be done in the following weeks to make things more bearable’ (participant 7)

**Supplementary data 12) - Colleague and senior responses following their experience of violence and aggression**

‘The trainers being more supportive and knowledgeable’ (participant 5)

‘I think if I had been better supported then it would have made a huge difference, I blamed myself at the time, i also found the job unmanageable afterwards, I was upset in the role, I felt detached and not present, I backed away from the team as a result and that lessened the support more (participant 7)

‘A peer group that’s embedded into the programme’ (participant 6)
